# Recurrent symptoms of acute intermittent porphyria after biochemical normalization with givosiran—An ongoing clinical conundrum

**DOI:** 10.1002/jmd2.12354

**Published:** 2022-12-13

**Authors:** Christopher D. Ma, Denise Faust, Herbert L. Bonkovsky

**Affiliations:** ^1^ Department of Internal Medicine, Section on Gastroenterology and Hepatology Wake Forest University School of Medicine Winston‐Salem North Carolina USA

## Abstract

A 47‐year‐old woman with acute intermittent porphyria (AIP) has had recurring symptoms after achieving biochemical normalization of her urinary 5‐aminolevulinic acid (ALA), porphobilinogen (PBG), and total porphyrins with givosiran. She has had normal liver tests, mildly decreased renal function, and sustained normal urinary ALA, PBG, and porphyrins with no rebound in her laboratory test results throughout treatment. She continues to tolerate monthly givosiran injections with no adverse effects, but she still experiences what she believes are acute porphyric attacks every 1–2 months.

## 1 INTRODUCTION

Acute intermittent porphyria (AIP) is an autosomal dominant condition caused by a mutation in hydroxymethylbilane synthase (*HMBS*), which can cause patients to experience neurovisceral and gastrointestinal symptoms. A biochemical hallmark of biochemically and clinically manifest AIP is marked up‐regulation of hepatic 5‐aminolevulinic acid synthase‐1 (ALAS1), the first and normally rate‐controlling enzyme of the heme biosynthetic pathway, which is under negative feedback control by heme, the end‐product of the pathway.^1^, ^2^ The treatment for acute attacks of AIP has been intravenous (IV) dextrose and heme, but in 2019, the US Food and Drug Administration and the European Medicines Agency approved the drug, givosiran, a siRNA that selectively down‐regulates ALAS‐1 expression in hepatocytes, to decrease the frequency of acute porphyric attacks.^3^ Givosiran has shown promising results in lowering the levels of urinary 5‐aminolevulinic acid (ALA) and porphobilinogen (PBG) while also decreasing the frequency of acute porphyric attacks.^4^, ^5^ Here we report a case of a patient with recurrent AIP symptoms despite receiving givosiran even after achieving persistently normal levels of urinary ALA, PBG, and total porphyrins.

### 1.1 Case report

A 47‐year‐old woman with a history of AIP with the HMBS mutation, c.839 G>A, p.Gly280Glu, a known pathogenic mutation,^6^ iron deficiency anemia due to menometrorrhagia, gastroparesis unrelieved by metoclopramide and erythromycin, and chronic kidney disease, presented to our Center for better management of her AIP. She had a 24‐year history of recurrent abdominal pain requiring many emergency department visits, local hospital admissions, and multiple surgeries, including a duodenal repair and a cholecystectomy, with no symptom relief. She was a former smoker and only rarely consumed alcohol. She had been diagnosed with AIP in 2008 at age 34 years, based upon the clinical picture, coupled with high urinary ALA, PBG, and total porphyrins. She was treated with IV heme (Panhematin, Recordati) through a central port; this treatment was complicated by a pulmonary embolism. Since then, she has refused to receive heme.

Prior to initiating givosiran, she used narcotics, promethazine, ondansetron, and IV dextrose for recurrent acute AIP attacks. She experienced acute AIP exacerbations almost monthly, typically after ovulation during the luteal phase of her menstrual cycles, and reported abdominal pain, generalized, stabbing neuropathy in her upper and lower extremities, fatigue, nausea, vomiting, and photosensitivity in sun‐exposed regions. She had used cimetidine 400 mg twice per day for 9 years and ranitidine 150 mg per day for 8 years but did not receive any relief from her AIP symptoms. At her initial visit to our center, her serum alanine aminotransferase was 11 U/L, aspartate aminotransferase was 19 U/L, alkaline phosphatase was 43 U/L, total bilirubin was 0.5 mg/dl, urinary ALA was 13.4 mg/g creatinine (Cr), urinary PBG was 29.6 mg/g Cr, and total urinary porphyrins were 648 nmol/g Cr (Table 1).

**TABLE 1:** Selected laboratory studies before and during givosiran treatment

Laboratory studies	Before Givosiran (4/2018)	Just prior to first dose (11/2018)	3 months after first dose (2/2019)	~1 year after first dose (12/2019)	~2.25 years after first dose (1/2021)	3 years after first dose (11/2021)	Reference Range
Serum ALT (U/L)	11	10	14	22	30	16	5–50 U/L
Serum AST (U/L)	19	15	22	38	51	30	5–40 U/L
Serum alkaline phosphatase (U/L)	43	50	61	69	80	59	25–125 U/L
Serum total bilirubin (mg/dl)	0.5	0.3	0.4	0.3	0.3	0.4	0.1–1.2 mg/dl
Serum albumin (g/dl)	4.2	4.1	4.1	3.9	4.4	3.8	3.5–5 g/dl
INR	‐	1.03	1.04	1.09	‐	1.12	0.8–1.1
Plasma homocysteine (μmol/L)	‐	‐	‐	‐	31.7	12.6	<15 μmol/L
BUN (mg/dl)	13	23	14	17	19	16	8–24 mg/dl
Serum creatinine (mg/dl)	1.13	0.96	1.06	1.33	1.07	1.06	0.5–1.5 mg/dl
eGFR (ml/min/1.73 m^2^)	53	> = 60	64	49	62	63	> = 90 ml/min/1.73m^2^
Urinary ALA (mg/g Cr)	13.4	‐	1.1	1.2	0.7	0.7	<7 mg/g Cr
Urinary PBG (mg/g Cr)	29.6	‐	2.6	1.4	0.7	0.8	<4 mg/g Cr
Urinary total porphyrins (nmol/g Cr)	648	‐	144	61	115	96	<300 nmol/g Cr

Abbreviations: ALA, 5‐aminolevulinic acid; ALT, alanine aminotransferase; AST, aspartate aminotransferase; BUN, blood urea nitrogen; Cr, creatinine; eGFR, estimated glomerular filtration rate; INR, international normalized ratio; PBG, porphobilinogen.

She tolerated well monthly injections of givosiran (2.5 mg/kg body weight). After 3 months of such treatment, serum aminotransferases remained normal, and urinary ALA, PBG, and total porphyrins had decreased to normal, 1.1 mg/g Cr, 2.6 mg/g Cr, and 144 nmol/g Cr, respectively. One year after the start of treatment, her urinary ALA was 1.2 mg/g Cr, urinary PBG 1.4 mg/g Cr, and her urinary total porphyrins were 61 nmol/g Cr (Table 1).

Although she achieved biochemical normalization in urinary excretions of porphyrins and their precursors, unfortunately, she continued to experience acute AIP attacks almost every month, usually during the luteal phase of her menstrual cycles. She stated that her symptoms mildly improved after the first few days of the monthly injections of givosiran, but she still reported increased chronic and postprandial abdominal pain, peripheral neuropathy, muscle aches, brain fog, fatigue, depressed mood, and insomnia. She required multiple further hospital admissions due to AIP attacks but continued to refuse IV heme because of her previous complication of pulmonary emboli. During those admissions, she was unable to eat due to severe postprandial abdominal pain, nausea, and non‐bloody, bilious emesis, and she was treated only with IV dextrose, hydromorphone, and ondansetron. She recently received bilateral splanchnic celiac plexus blocks performed by a pain specialist, which, she reported, improved her pain by approximately 90%.

With ongoing monthly givosiran and management by the specialist in the management of chronic pain, she has had fewer acute attacks, but she continues to suffer what she considers typical acute porphyric attacks every 4–6 months, despite persistently normal urinary ALA, PBG, and total porphyrins.

## 2 DISCUSSION

Givosiran is a siRNA that targets hepatic ALAS1 to downregulate the production of ALA, PBG, and total porphyrins in the heme biosynthesis pathway.^3^ The pathogenesis of acute porphyric attacks remains incompletely understood. Most persons with known pathogenic mutations in *HMBS* never experience any attacks; others, mainly young women, experience only one or a few rare attacks, especially if the correct diagnosis is made and if they avoid known triggers of such attacks. The cardinal symptoms of AIP attacks, which include gastrointestinal and neurovisceral symptoms, are likely induced mainly by elevated ALA and/or products derived therefrom, and perhaps PBG, which can cross the blood–brain barrier and exert neurotoxic effects throughout the central, peripheral, and autonomic nervous systems.^1^, ^7^ ALA is structurally similar to gamma‐aminobutyric acid (GABA), an inhibitory neurotransmitter in the central nervous system, and can interact with GABA receptors by acting both as a partial agonist and an antagonist.^2^ High levels of ALA in patients with AIP can induce oxidative stress and influence other neurotransmitters, like glutamate, causing neurological symptoms, such as seizures, delirium, and confusion.^2^, ^8^ When these intermediates are lowered with givosiran or heme, it is thought that the frequency and severity of acute attacks are ameliorated. Our patient responded promptly to givosiran with normalization of levels of urinary ALA, PBG, and porphyrins (Table 1), but continued to experience frequent attacks of abdominal pain and other symptoms that she interpreted as continuing typical acute porphyric attacks, albeit with a lower frequency than prior to the start of givosiran.

Givosiran has usually been well‐tolerated without serious adverse effects and has been reported to significantly decrease the mean annualized rate of composite porphyric attacks and decrease supplemental hemin use by 83% and 68%, respectively, in a long‐term continuous givosiran group in a 24‐month interim analysis of the ENVISION study.^5^ Nevertheless, there are adverse effects, such as rare severe allergic reactions, elevations in liver tests, which may require cessation of use (Ma & Bonkovsky, unpublished observations), decreases in renal function, and elevations in plasma homocysteine levels.

This patient reported more frequent porphyric attacks during her menstrual cycles, specifically around ovulation, which is a common finding among women with AIP. Estrogen and progesterone induce ALAS‐1, increasing the levels of porphyrin intermediates, which play a role in causing acute attacks.^9^, ^10^ This is further supported by results of a recent study that used an in vitro drug fluorescence screening assay to test for porphyrogenicity; both β‐estradiol and norgestrel, drugs in the estrogen and progestogen classes, respectively, led to a significant accumulation of protoporphyrins.^11^ Our patient continued to have low ALA and PBG even during her menstrual cycles, so it is still unclear what the etiology and pathogenesis are for her ongoing symptoms. Perhaps, she has local neuronal ALA excess or relative heme insufficiency in chronically damaged and compromised nerve cells. She responded well to bilateral splanchnic celiac plexus blocks, which alleviates abdominal visceral pain, so if patients like her continue to experience frequent acute attacks while taking givosiran, despite normalization of the over‐production of ALA and PBG, alternate forms of pain management, including nerve blocks, should be considered. Patients who do not achieve normalization of ALA and PBG plasma or urinary levels might be considered for trials of givosiran dose escalation. While on treatment with givosiran, our patient developed transient mild hyperhomocysteinemia, which resolved spontaneously without discontinuation of givosiran or the use of pyridoxine or other B vitamins, and a transient decrease in her estimated GFR. A decrease in GFR in response to givosiran is a well‐documented adverse side effect.^4^ The association of givosiran and transient hyperhomocysteinemia is recognized with increasing frequency^12^, ^13^, ^14^ and has been associated with variants of the methylene tetrahydrofolate reductase gene.^15^

In summary, we report a patient with AIP who experienced biochemical resolution of her porphyrin studies with monthly givosiran injections yet continued to experience AIP symptoms. Overall, this case highlights the importance of careful ongoing clinical observations of responses to medications and the possible benefits of implementing other treatments, like splanchnic nerve blocks as in our patient, to optimize patient care.

## AUTHOR CONTRIBUTIONS

Christopher D. Ma and Herbert L. Bonkovsky conceptualized the project, completed the chart review, and wrote the manuscript. Denise Faust assisted with the chart review and reviewed the manuscript. All authors have read and approved the submission of the final manuscript.

## CONFLICT OF INTEREST

The author declares that there is no conflict of interest that could be perceived as prejudicing the impartiality of the research reported.

## Data Availability

The data that support the findings of this case report are available from the corresponding author upon request.
